# The Public Health Response to COVID-19 in Vietnam: Decentralization and Human Rights

**DOI:** 10.1007/s41649-022-00226-1

**Published:** 2022-10-22

**Authors:** Hai Thanh Doan

**Affiliations:** Bioethics Centre, University of Otago, Dunedin, New Zealand

**Keywords:** Human rights, Public health, Vietnam, Decentralization, COVID-19

## Abstract

Human rights constitute a universal concern in different countries’ responses to COVID-19. Vietnam is internationally praised for its success in containing the pandemic; nevertheless, human rights issues are a key area that needs to be assessed and improved. Little legal and ethical research is available on human rights in Vietnam, particularly in its response to COVID-19, however. In Vietnam, decentralization took place during the pandemic: higher authorities delegated power to lower ones to make and implement public health measures. Unfortunately, many measures made and implemented decentrally caused human rights concerns or breaches. This article aims to study what makes such measures cause human rights concerns or breaches. It argues that several social, legal, and political factors, including an inadequate understanding of human rights, the undefined breadth of discretion, and lack of supervision, are underlying factors for such problematic decentralized measures. Accordingly, this paper proposes two solutions (i) improving the supervision of the decentralization process, and (ii) improving the understanding of human rights. While Vietnam should learn from the international community to improve its measures, lessons and experience from Vietnam can also contribute to a richer dialogue and better protection of human rights globally.

## Introduction

Globally, human rights have been overlooked and vulnerable during the COVID-19 pandemic (Daȩbrowska-Kłosińska 2021; Desierto 2020, 2021; de Mesquita et al. 2021). In different countries, this global problem takes nuanced shapes—though, with close investigations, some common patterns might be found (ten Have 2022). It follows that studying different countries not only helps these countries to spot their problems and find solutions but also helps the global community to understand some common mistakes. Internationally, Vietnam is praised for its success in containing the pandemic, especially in the early stages (Ivic 2020; Pollack et al. 2021; Quach et al. 2021; Thao and Đào 2021). Notwithstanding, its approach is not spotless. Human rights issues are the key area to be assessed and improved. However, the literature studying this issue, especially being informed by in-depth Vietnamese background, is still unsatisfactory.

In Vietnam, decentralization took place during the pandemic: higher authorities delegated power to lower ones to make and implement public health measures. Unfortunately, many measures made and implemented decentrally caused human rights concerns or breaches. This article aims to study what makes such measures cause human rights concerns or breaches. These questions shall be addressed in the below five sections.

This paper first studies three controversial Vietnamese cases concerning public health measures in “Cases”. Actually, controversial cases are more than three, however, due to the constraint of space, only three notable cases are retold. The reason for retelling these three cases is that first, these caused wide public outcries. Second, either the Government or local authorities recognized problems concerning these—though they might not do so explicitly or recognize that human rights were breached. By retelling cases, this paper gives a sense of how controversial some social public health measures might be. In “What Human Rights Are at Stake?”, from the three cases retold, then the paper goes on to figure out what human rights are at stake in each case. Three cases are also demonstrative of how measures have been made decentrally. Due to the constraint of space, I shall not retell in detail how, procedurally, measures were made and implemented decentrally. In “The Need for Decentralization”, I explain why in opposition to the observation that centralization does better in managing the pandemic (ten Have 2022), in Vietnam and, perhaps, many other countries, there are reasons for decentralization. In “Why does Decentralization go Wrong?”, this paper offers a contextualized account of what makes such measures cause human rights concerns or breaches. It argues that several social, legal, and political factors, including an inadequate understanding of human rights, the undefined breadth of discretion, and lack of supervision, are the underlying factors for such problematic decentralized measures. Finally, this paper proposes two solutions to prevent or minimise human rights concerns or breaches (i) improving the supervision of the decentralization process, and (ii) improving the understanding of human rights.

## Cases

### Case 1: ‘Bread is not Food’

On 18 July 2021, there was a video circulating widely on online platforms, including YouTube, Facebook’s groups and pages, and online forums. The video of about 3 minutes recorded a situation in which a man claiming himself as the Deputy Chairman of Vinh Hoa Ward in Nha Trang, also chair of the ward’s COVID-19 prevention committee and officers of the ward Cordon Sanitaire for pandemic control booked E, a worker of a local public construction project, for riding his motorbike during social isolation. At that time, there was nobody on the street except for E, the Deputy Chairman, and officers. E claimed that he had just gone from the construction site to buy bread and water and been able to show a ‘work confirmation letter’ stamped by his company as required by Directive 16; E argued that he should be allowed to go out for food. However, the Deputy Chairman and officers rejected E’s argument, yelling that “bread is neither staple food, e.g. rice, nor [raw] food; the purchases of Ya are non-essential’ (*Tuổi Trẻ* 2021a). The Deputy Chairman then decided to seize E’s motorbike registration, driver’s license, and vehicle (*Tuổi Trẻ* 2021a), requested him to come to the Ward to pay the fine, and pressured E’s employer to fire him for daring to argue [against the Deputy Chairman]. E was fired by his employer immediately on the same day. It later came to light that the Deputy Chairman had previously fined bakeries for making bread, also arguing that it was unessential (Long 2021).

On the same day, the case was reported widely in state-backed media or newspapers and a subject of debate and provoked public outcries as to whether such sanctions are grounded. Also on the very same day, in responding to questions from reporters about the video, the identity of the man claimed as the Deputy Chairman of Vinh Hoa Ward, and his behaviour, the Chairman of the People Committee of Nha Trang City confirmed the identity of the Deputy Chairman of Vinh Hoa Ward and endorsed the fine (though he did not explicitly state the same reason that the Deputy Chairman of Vinh Hoa Ward articulated in the video), stating that:

Nha Trang City is in the course of implementing Directive 16… Currently, restaurants, diners and non-essential businesses in the area are closed; even takeout is prohibited.

For food supply, the city had previously developed a plan to issue shopping permit cards (thẻ đi chợ) which permit people to go to the market once every 3 days.

…

It is illegal for Mr E to go out to buy bread and drink water. At the same time, the inspection team … has an inappropriate attitude (Chiến 2021).

In short, the Chairman of the People Committee of Nha Trang City might imply that what was only wrong was the rude attitude of the Ward’s Depute Chairman but not his decision.

The position of the Deputy Chairman of Vinh Hoa Ward only received minor support from Force-47 groups and extremist individuals heavily influenced by discourses of Force-47 groups. The justification for the measure of Vinh Hoa Ward and Nha Trang City is that if citizens can freely go out to buy food, they can pretend to buy food to go out and walk around. Consequently, the policy aim of the measure shall fail should any loopholes be exploited. However, the majority of the population did not accept the Deputy Chairman of Vinh Hoa Ward’s behaviour and justification.

In response to public outcries, on the morning of 20 July, the Standing Committee of Khanh Hoa Province had a meeting with the Steering Committee for disease prevention and control of Khanh Hoa Province and that of Nha Trang city. After listening to the Report of Nha Trang City People’s Committee, the Standing Committee of Khanh Hoa Province opined that:

…on the incident of Vinh Hoa Ward People’s Committee…, Vice Chairman of Vinh Hoa Ward People’s Committee was inadequately understood, leading to rigid and improper implementation of the Prime Minister’s Directive No. 16/CT-TTg…; had an improper attitude and behaviour towards the people while carrying on public duties, causing public outcries…

The Standing Committee of Khanh Hoa Province requested Nha Trang City’s Party Committee and People's Committee to continue … verifying … the case, reprimanding … relevant officials in accordance with regulations, and publicizing information for the people to know and supervise. (Báo Điện tử Chính phủ 2021)

Following that the Chairman of the People Committee of Nha Trang City issued a letter recognizing that ‘the Deputy Chairman of Vinh Hoa Ward was misunderstanding the Directive’ and apologised to E on behalf of agencies of Nha Trang City (Toàn 2021). Then, the Deputy Chairman of Vinh Hoa Ward publicly apologised to E. The Government’s Facebook page (‘Thông tin Chính phủ’) and the Government News Portal also publicly updated about the apology process, including the letter of the Chairman of the People Committee of Nha Trang City and the personal apology from the Deputy Chairman of Vinh Hoa Ward (Báo Điện tử Chính phủ 2021).

### Case 2: Coercing to Take a COVID-19 Test Administered by Public Officers

The second case is related to a woman physically forced to take a COVID-19 test administered by public officers. At the end of September 2021, in a video circulating on YouTube and Facebook’s groups and pages, a woman was teaching an online yoga class at her apartment when someone knocked on the door and forced her to leave her room to get a COVID-19 test administered by public officers. The woman replied that she had already conducted a COVID-19 rapid antigen test (RAT) by herself, adding that she did not want to come into contact with others and that she was in the middle of teaching a class (*Tuổi Trẻ* 2021b). Then, a group of officials, police officers, and members of the apartment complex’s management board headed by the Party Secretary of Vinh Phu Ward’s Party Committee cut the door lock, coerced, and escorted her to the building’s courtyard for a nasal swab collection amidst her babies’ cries.

As soon as the video was circulated, public outcries broke out. In response to public outcries, the representative of the official team noted that the woman’s apartment was exposed to high risk as infections were detected in the block (*Tuổi Trẻ* 2021b). Then in a press conference following the event, to justify the measure implemented by the Party Secretary of Vinh Phu Ward, Thuan An’s City Party Secretary argued that ‘at a time when the whole political system is straining itself against the COVID-19 pandemic, all laws cannot be applied normally…’ (Dũng 2021). Notwithstanding, ultimately, the Secretary of Vinh Phu Ward’s Party Committee apologised to the woman. There was no further remedy though.

### Case 3: Culling Dogs

The third case concerns a situation in which 13 dogs belonging to a married couple were culled in the middle of October 2021. After dogs’ owners tested positive for SARS-CoV-2, Tran Van Thoi District’s health authorities figured out that dogs’ fur had abnormal substances and decided to cull them, fearing the animals carried the virus (*Tuổi Trẻ* 2021c). The news indicated that there lacked clear evidence that further investigation or measures were considered or implemented. The killing has stirred up an uproar on Facebook where people criticized the decision for being unscientific and cruel (*Tuổi Trẻ* 2021c).

In a press conference following the event, the Chairman of the Tran Van Thoi District People Committee admitted that “the animals should have been culled only when they had been confirmed to be infected with the disease”; nevertheless, he argued that such a solution is unfeasible given the pressure and limited capability: “If we had had better management capacity, we could have isolated the pets, disinfected them, and monitored them carefully”(*Tuổi Trẻ* 2021d). In the prevention and control of COVID-19, [due to lacking resources] ensuring people’s health and preventing cross-infection in quarantine facilities would be the top priorities; the culling, hence was necessary, he concluded (*Tuổi Trẻ* 2021d). The Chairman of the Tran Van Thoi District People Committee also claimed that officials had sought consent from dogs’ owners. Notwithstanding, dogs’ owners claimed that authorities did not seek consent in advance.

## What Human Rights are at Stake?

In this section, I shall discuss what human rights are at stake in each case. I shall use the term ‘human rights concerns’ and ‘may violate human rights’ to signify that what is discussed here is in no way conclusive. The reason for this is that while the terminology ‘human rights’ can be nuanced when it is used in different fields, law, philosophy, or ethics…, the term ‘human rights violations’ is of strong legal connotation and must be defined with clarity, certainty, and accuracy by referring to a set of legal standards. Specifically, to assess whether measures that restrict human rights violate any rights, it is necessary to assess whether such measures comply with the Siracusa principles.^1^ As the Siracusa principles also recognize the pluralism of models of a democratic society and socio-economic status among countries… a practice can be a violation in one jurisdiction but not in another jurisdiction. It would follow that asserting a practice as having violated human rights can sometimes be not straightforward but very dedicated, especially in contentious bioethics and health issues.^2^ Though the 2013 Constitution, for the first time in the history of the Socialist Republic of Vietnam, articulates explicitly the protection of human rights under Article 3,^3^ recognizes the applicability of the Siracusa principles under Article 14,^4^ and dedicates a whole chapter (Chapter II) for human rights, a Vietnamese jurisprudence on human rights issues and specific judgments or official statements in this regard are still absent. To claim and demonstrate how some public health measures are problematic and may violate human rights, I refer to articles of ICCPR, ICESCR, and Vietnamese Constitutions in conjunction with the jurisprudence of other jurisdictions. Also, this paper shall not engage with legal consequences of the declaration of public health emergencies here (including implications it has on human rights) since Vietnamese Prime Minister Phạm Minh Chính announced clearly that after careful consideration, the State of Emergency would not be declared (though he admitted that emergency measures have already been implemented) (Khuyên 2022; Long and Lê 2021).^5^

### Case 1: the Right to Freedom of Movement

The right to freedom of movement (as opposed to the right to liberty and security) is relevant as the measures at hand did not ban all movements: public offices and construction sites were allowed to open and operate, and citizens were allowed to leave their homes once per three days to buy food.

Pursuant to Article 13 of UDHR and Article 12 of ICCPR ‘Everyone lawfully within the territory of a State shall, within that territory, have the right to liberty of movement…’ (see same is prescribed under Article 2 of Protocol 4 to the ECHR, Article 12 of African Charter on Human and Peoples’ Rights, Article 22 of American Convention on Human Rights, Paragraph 15 of ASEAN Human Rights Declaration, Article 23 of the 2013 Constitution).

Though Vinh Hoa Ward’s measures vindicate a legitimate objective that is the protection of public health and proponents of such measures have tried to argue that strict measures are practically necessary because as long as citizens can freely go out to buy food, they can pretend to buy food to walk around; consequently, there were loopholes to be exploited. Notwithstanding, the measures can be problematic from the view of human rights law. Though the pandemic is an existing grave threat, the measures express an aim to curb the spread of the virus, and it is a consensus in every legal system (at the national, supranational, and international levels) that authorities should enjoy the margin of appreciation (Evans v. the United Kingdom at para. 77, Koufaki and Adedy v. Greece at para. 37, Vavřička and Others v. The Czech Republic at para. 274–275), Again, to be legally legitimate, restrictions must satisfy the Siracusa principles with which measures of Vinh Hoa Ward seem to fail to satisfy.

*First, all restrictions must always have a legal basis*. Pursuant to General Comment 27 on freedom of movement, ‘The law has to establish the conditions under which the rights may be limited. Restrictions which are not provided for in the law would violate the right to freedom of movement’ (para. 12).

In the case of Vietnam, there was no law banning movement for essential needs like buying food. The law on Prevention and Control of Contagious Diseases (LPCCD) regulating the measures that can be taken by the authorities to prevent and control contagious diseases with human-to-human transmission provides a range of measures for the prevention and control of infectious diseases under Sect. 3 (from Articles 46 to 56 of LPCDD^6^). Social intervention measures are provided under Articles 49 to 55 of LPCDD. The two most notable provisions are Article 53 on control of entry into and exit from class-A epidemic zones (Under Article 3.1 of LPCDD class A consists of extremely dangerous infectious diseases that can transmit very rapidly and spread widely with high mortality rates or with unknown agents) and Article 54 dealing with a state of emergency in case of an epidemic.

Pursuant to Article 53, measures for controlling entry into and exit from zones infected with class-A epidemic diseases include (i) restricting persons and means of transport from entering and leaving epidemic zones; in case of necessity, medical inspection, surveillance, and disposal shall be conducted; (ii) prohibiting transportation from epidemic zones of articles, animals, plants, food, and other commodities capable of transmitting the epidemic disease; (iii) taking personal protection measures, for persons entering epidemic zones specified in Clause 1, Article 51 of this Law; (iv) Other necessary measures as prescribed by law.

Pursuant to Article 54, when declaring a state of emergency in case of an epidemic, the head of the steering committee has the following powers: (i) mobilizing and requisitioning resources specified in Article 55 of this Law; (ii) placing signboards, guard stations, and instructions on travel bypassing epidemic zones; (iii) requesting medical inspection and disposal of means of transport before they leave epidemic zones; (iv) prohibiting mass gathering and other activities likely to transmit the epidemic disease in epidemic zones; (v) prohibiting persons and vehicles from entering epidemic foci, except for those on duty; (vi) conducting disinfection and sterilization on a large scale; (vii) culling animals and destroying food and other articles likely to transmit the epidemic disease to humans; (viii) taking other measures.

On its face, although Article 53 and Article 54.2.d, 54.2.e of LPCDD can be interpreted as permitting a lockdown over large regions via defining the epidemic zones to the broadest possible extent, e.g. a province, groups of provinces, or even the whole nation, overtly restricting most sorts of movements, including movements to buy food, seems to be problematic. The permission to take any ‘other necessary measures’ can also be used to justify Vinh Hoa Ward’s measures though this permission seems to be problematic due to lacking well-defined regulatory breadth and criteria for restricting human rights. Notwithstanding, in practice, measures of local authorities did not invoke the LPCCD but Directive No. 16/CT-TTg dated 31 March 2020 (Directive 16) of the Prime Minister instead. This Directive stipulated that all facilities except essential ones^7^ were forced to be closed. Gatherings of more than two persons in public places were prohibited. Directive 16 is not law or bylaw regulation, however. Pursuant to Article 30 of the Law on Organizing the Government:

1. The Prime Minister shall promulgate legislative documents within his/her jurisdiction so as to perform his/her duties and powers, inspect the implementation of such documents and deal with documents in breach of the Constitution and legislation.

2. The Prime Minister shall act on behalf of the Government to sign the Government’s documents; issue decisions, directives and instructions, and examine the implementation of such documents in state administrative organs at the central level through the local one.

This means the scope of application of Prime Minister’s Directive 16 was internal to the Government’s structure. It is not a legislative legal document that can be applied widely to the citizenry. Indeed, the former Prime Minister Nguyễn Xuân Phúc who passed Directive 16 did claim that it was not a legal document and not binding… (Tuân 2020). Moreover, restricting the movement for buying food (i.e. bread) seems also not to be aligned with this Directive.

*Second, the laws authorizing the application of restrictions should use precise criteria and may not confer unfettered discretion on those charged with their execution* (General Comment 27, para. 12). Measures of Vinh Hoa Ward are problematic because defining what is ‘essential’ is at the unfettered [and unreasonable] discretion of those in charge of execution. It is hard to think why authorities of Vinh Hoa Ward allow indoor public offices and construction sites to open but ban the making of bread and movements to purchase bread. It is against common sense that authorities of Vinh Hoa Ward claim that bread is not food. The power [to define what is essential] was observable conferred unfetteredly and misused by *the* authorities of Vinh Hoa Ward.

*Third, the measure at stake must be (i) necessary, (ii) appropriate to achieve its protective function, (iii) conform to the principle of proportionality, and (iv) the least intrusive instrument among those which might achieve the desired result*. Though restricting movement does help cut the chain of infections, it is not clear if a blanket ban of almost almost all activities like in this case satisfies the requirement of proportionality and is the least intrusive measure. Arguments of proponents of Vinh Hoa Ward’s measures are invalid because as asserted by public health experts of the Government, the purpose of the policy is not about banning or restricting movements but to protect public health (VOV 2021); hence, the implementation of measures should not be about trying to ban all movements or as many movements as possible.

In Communauté Genevoise D’action Syndicale (CGAS) v. Switzerland, the majority of ECtHR ultimately concludes that the COVID-19 measure of Switzerland was disproportionate in light of a number of factors, including the importance of freedom of assembly in a democratic society, the long duration of the absolute prohibition, the fact that a range of other activities (including indoors) remained permitted under the relevant COVID-19 regulations, and the threat of criminal sanctions (Smet 2022).

In the Vietnamese case, the sanction against movements was less coercive; however, the importance of finding subsistence, the fact that a range of indoor activities remained permitted, and the sine die nature of the measures at that time should be taken into account to find a possible breach of the right to freedom of movement.

### Case 2: The Right to Life, Liberty, and Security of the Person, and the Right to Respect for Privacy, Family, Home, and Correspondence, and Protection of Honour and Reputation

The right to life, liberty, and security of person (Article 3 of UDHR, Article 9 of ICCPR, Article 3 of the ECHR, Article 6 of African Charter on Human and Peoples' Rights, Article 7 of American Convention on Human Rights, Paragraph 12 of ASEAN Human Rights Declaration, Article 20 of the 2013 Constitution) and right to respect for private and family life (Article 12 of UDHR, Article 17 of ICCPR, Article 8 of the ECHR, Article 11 of American Convention on Human Rights, Paragraph 21 of ASEAN Human Rights Declaration, Article 21 of the 2013 Constitution) can be the relevant rights in this case.

Article 3 of UDHR, Article 9 of ICCPR, and equivalent articles under other conventions provide for the protection of three separate but related rights that are the right to life, the right to liberty, and the right to security of person. It needs to make clear that the case at hand relates to the right to security of a person (but not the right to life and the right to liberty). Security of a person concerns freedom from injury to the body and the mind, or bodily and mental integrity (General Comment No 35, para. 3). State agents must refrain from treatment which damages a person’s physical health or causes them mental or psychological harm (European Court of Human Rights 2015).

Article 12 of UDHR, Article 17 of ICCPR, and equivalent articles under other conventions provide for the protection of some interrelated rights which are the right to respect for privacy, family, home, correspondence, and protection of honour and reputation. The right to respect for privacy, family, home and correspondence, and protection of honour and reputation is required to be guaranteed against all arbitrary interferences and attacks whether they emanate from State authorities or natural or legal persons (General Comment No 16, para. 2). States are under both positive and negative obligations to secure the right to effective respect for physical and psychological integrity. In any event, measures should be reasonable in particular circumstances. Compliance should be guaranteed de jure and de facto. Even concerning interferences that conform to the Covenant, relevant legislation must specify in detail the precise circumstances in which such interferences may be permitted. A decision to make use of such authorised interference must be made only by the authority designated under the law, and on a case-by-case basis (General Comment No 16, para. 8). Free and informed consent to medical treatment is under the auspice of this right.

In South Africa, compelled testing is generally not permitted, except when supported by a court order. In the case of C v Minister of Correctional Services (1996), informed consent as a necessary pre-requisite to HIV testing was reaffirmed, with pre-counselling forming a key part of the process to obtain such informed consent. Meanwhile, under the jurisdiction of ECtHR, the Court found that relatively minor medical tests, which are compulsory (see Acmanne and Others v. Belgium, Commission decision; Boffa and Others v. San Marino, Commission decision; Salvetti v. Italy (dec.)) or authorised by court order (X v. Austria, Commission decision; Peters v. the Netherlands, Commission decision), may constitute a proportionate interference with Article 8, even without the consent of the patient. Within the context of Vietnam, Article 21 of LPCCD on the contents of infectious disease surveillance provides a ground for mandatory testing, accordingly ‘…In case of necessity, competent health agencies may take testing samples from persons suspected of suffering infectious diseases for supervision.’

Vietnamese law does not require an order from a court or equivalent bodies for mandatory testing. This may pose constitutional questions as to whether orders should be required for mandatory testing [by the law]. Besides, the terms used such as ‘necessity’ or ‘suspected’ are not well explained which can result in the endowment of unfettered discretion on those charged with their execution. Notwithstanding, because there is evidence that buildings that have heating, ventilating, and air conditioning (HVAC) systems but the systems do not exchange old air for fresh air can be all at risk of infection of COVID-19 (MoH 2022; CDC 2021; McKee 2021), the level of risk and urgency of testing should be recognized. However, what is contested in this case is mandatory testing and mandatory taking a test administered by public officers in public space need not be the same. A mandatory taking RAT together with a tele-supervision can be less intrusive and reach the same policy target. Besides, it is doubtful if packing people in a dense environment to force them to get a COVID-19 test administered by public officers while medical tools, e.g. medical gloves, are in shortage do help prevent and control diseases or may worsen the situation. Moreover, a legal provision that prescribes mandatory testing does not give a ground for coercive testing by breaking into homes or using force. Bearing these considerations in mind, Vinh Phu Ward’s measures can be disproportionate. Depending on the assessment of whether the interference with the body and private space reaches the threshold of damaging a person’s physical health or causing them mental or psychological harm, Vinh Phu Ward’s measures may violate either or both the right to life, liberty, and security of person and the right to respect of privacy, family, home, and correspondence, and protection of honour and reputation.

### Case 3: the Right to Property

This case relates to the right to property (see Article 17 of UDHR, Protocol 1 to the ECHR, Article 14 of African Charter on Human and Peoples’ Rights, Article 21 of American Convention on Human Rights Paragraph 17 of ASEAN Human Rights Declaration). The 2013 Constitution particularly stresses the right to private property (Article 23, Article 32, Article 51).

Vietnamese law provides a ground for culling animals provided that such animals are vectors of transmission of diseases to humans or other animals (see Article 50, 54.2.g of LPCCD, Article 25 of Law on Veterinary Medicine). Article 46 of LPCCD prescribes that an anti-epidemic steering committee has the tasks of taking anti-epidemic measures and overcoming epidemic consequences, and setting up mobile anti-epidemic teams to directly render first aid, provide medical treatment, and deal with epidemic foci. Reading 46, 50, 54.2.g of LPCCD allows an interpretation that (i) there is a legal foundation for culling, and (ii) the power of ordering culling of ‘infected’ animals is under the authority of the chairman of the Tran Van Thoi District People Committee, public health officials and veterinary stations. What is required next is to assess whether the culling activity is proportionate and if any other measures are less restrictive and intrusive. In assessing the proportionality, the [urgency of the] context, the [limited] resources at stake, and the margin of appreciation should also be taken into account. Even if the proportionality condition is satisfied, to determine the legality of measures, procedural matters, i.e. seeking informed consent and compensating should be factored in. In this case, there is no clear evidence that before culling dogs, other less intrusive measures have been considered and appraised (the Chairman of the Tran Van Thoi District People Committee justified this on the ground of urgency), that informed consent has been collected, and that sufficient remedy has been provided.

## The Need for Decentralization

Contrary to some claims of bioethicists as to how centralization might be more effective to handle emergencies (ten Have 2022), from the above cases, it is clear that many measures have been made and implemented decentrally, primarily by commune authorities. The question is why commune authorities have been put in the driving seat of making [such controversial] policies.

Actually, contrary to bioethicists’ observations, the fact that decentralization has taken place is hardly surprising. It is a conventional shared belief that amidst emergencies, power needs to be decentralized and executive power needs to be deferred in response to the demand for swift, decisive, effective, and appropriate decisions (Dung 2021; Giao and Đức 2021; Locke 1967). Indeed, the Vietnamese Government recognized this conventional shared belief. It claimed that decentralization in public health decision-making and implementation facilitate timely and innovative decisions that take into account the specific local context and avoid bureaucratic delays (Nguyen 2021). Indeed, over-centralization can be slow and disconnected from the social context of different localities: it is not only that the spread of diseases is different among regions but it is arguably true that 1000 infectious cases detected in populous cities can be more alarming than 1000 infectious cases in countrysides. Besides, centralized decisions, predictably, might cause uneven damage to different localities. For populous and large countries, decentralization somehow is unavoidable: it is more accurate to say that population and geographical factors allow effective centralization takes place than centralization seems to be more efficient than decentralization. It would follow that for many countries, there are reasons for decentralizing and decentralization being institutionalized as a response to disease outbreaks. The LPCDD recognizes this. Article 46 of LPCDD requires that when an outbreak of a contagious disease is declared, the central government and the local governments establish steering committees for the prevention and control of contagious diseases [the steering committee(s)]. The steering committees are bodies in the driving seat of organizing the enforcement of public-health measures (Article 46.3).

Another reason that explains why decentralization took place is that given the context of the pandemic or public health emergencies, making a centralized policy-making process is like carrying a mountain of responsibility on the shoulder. Typically, the centralized policy-making process must opt for either millions of lives or millions of dollars (and hence, again millions of lives). Any miscalculation may cost too much for a whole nation. This is significantly stressful, both in terms of morality and political responsibility. Making decisions is never easy; making decisions that can affect millions of lives, especially in a situation where millions of lives have been lost, is traumatic. As such, authorities might incline to delegate their power to a lower office to take down burdens of moral and political responsibilities. Decentralization, as such, can be used as a tool to mask and be shielded from moral and pollical responsibilities. In reality, the central government delegated power to provincial authorities. Provincial authorities then passed documents which read that the city or district (city) authorities had an obligation to make and implement measures and be responsible for such measures. In turn, the city authorities delegated power and responsibility to commune or ward (commune) authorities.

## Why does Decentralization go Wrong?

From these cases, it can be observed that many problems happen with decisions made and implemented decentrally, especially by commune authorities. The question then is why commune authorities—or other local authorities—may commit human rights breaches. This paper argues that several social, legal, and pollical elements are underlying factors, including poor understanding of human rights, the undefined breadth of discretion, and lack of supervision.

### Inadequate Understanding of Human Rights

Even in the scenario of centralization, as the implementation of measures needs to respect human rights, an adequate understanding of human rights matters a lot for public health measures. It is even more important in the scenario of decentralization.

Unfortunately, on the one hand, human rights have been cast into doubt constantly during the pandemic. Observing how ‘ambitious’, ‘stringent’, and ‘aggressive’ measures deployed by the Chinese Government in the first wave of the COVID-19 outbreak in Wuhan can be super effective, gives credit to criticism against human rights as to how democratic principles and human rights are irrelevant and burdensome in ‘the war’ against the pandemic (Huang 2020). Such kind of sentiments can be found flooded in Chinese and Vietnamese social networks and also in narratives of decision-makers, such as Thuan An’s City Party Secretary, for example.

On the other hand, it should be noted that for a while, human rights have not been purely legal but also politicized and political. The Conservative wing in Vietnamese society and public offices is quite hostile against human rights, seeing the discourse of human rights as a mere tool of colonialism and interventionism, or put it differently, the discourse of human rights has been exploited by hostile forces (‘thế lực thù địch’) and monopolized by foreign forces to criticize governmental affairs and fuel internal disorders, thereby making justifications for external interventions. Human rights have long been cast negative light and distorted. Meanwhile, human rights education and training have been inadequate. Only recently, on 5 September 2017, the Vietnamese Prime Minister issued Decision No. 1309/QD-TTg, approving the Scheme to incorporate human rights content into the national education system’s curricula. Still, materials on human rights education and training, including core international instruments, have not been adequate (Giao and Tùng 2008; Minh 2021).

As this paper will demonstrate, with the undefined breadth of discretion and the lack of supervision, it is not surprising that local authorities who have not been equipped adequately with human rights knowledge or even held hostility against human rights might breach human rights. Even for officers who are not charged with and capable of inventing measures, inadequate understanding of human rights may result in just-following-orders acts which are, as shown in ‘Eichmann in Jerusalem’ (Arendt and Kroh 1964).

### The Undefined Breadth of Discretion: Socialist Legality

Under Article 46.3 of LPCDD, the steering committees on prevention and control of diseases are bodies in the driving seat of organizing the enforcement of public-health measures and under Article 53 and Article 54 of LPCDD, chairs of the steering committees are capable to take any ‘other necessary measures’, read these provisions together, it is not clear whether LPCDD vests in the steering committees only the power to implement measures or both the power to make and implement measures. In the latter case, the question is how far the breadth of discretion to ‘invent’ measures is. It seems that the breadth of discretion is not well-defined and not aligned with the 2013 Constitution and the Siracusa principles.

Socialist legality might be the primary reason for the undefined breadth of discretion. Since the 1950s, the Soviet legal-politico traditionhas been embraced in Vietnam (Nghia and Ha 2018). A key pillar of Soviet legal-politico theory is the ‘socialist legality’ (pháp chế xã hội chủ nghĩa). According to ‘socialist legality’, the law is a mere instrument to concretize the control over the means of production of such a ruling class, reflecting the ‘will of the ruling class’ (ý chí của Giai cấp thống trị) (Duẩn 1980; Tâm 2009). It is not difficult to see that ‘socialist legality’ provides a ground for the undefined breadth of discretion.

*First,* in light of *socialist legality,* all other constitutions, except for the 2013 Constitution, allow fiat to restrict human rights if deemed necessary. Provisions of LPCDD that endow local officers' undefined breadth of discretion are an expression of *socialist legality* and are also in line with the 1992 Constitution (Dung 2021).

*Second,* in light of *socialist legality*, the law can be elastic, depending upon the state’s mind. Consequently, cogent legal reasoning or justifications for discrepancies in policies and measures are not obligatory and deemed as not necessary. For example, in 2020, then-Prime Minister Nguyễn Xuân Phúc interpreted Directive 16 as neither a lockdown nor a traffic ban that would result in ‘blocking the river or prohibiting markets’ (ngăn song cấm chợ) and warmed against divergent, inconsistent, and excessive measures of local authorities (Anh 2020; Tuân 2020); however, in light of the Delta wave, several official dispatches and telegrams from the Government, e.g. Official Telegram 1099/CĐ-TTg, 1102/CĐ-TTg, permitted and encouraged provinces to put measures that prevent citizens from leaving their homes and flexibly ‘invent’ and implement measures. The Government also required that local authorities’ measures must be ‘one-level higher’ and ‘one-step earlier’ than the measures of the Government (*Tuổi Trẻ* 2021e).

### Lack of Supervision

#### Unclear Chain of Command

Pursuant to Art. 46 of LPCDD, the national steering committee on prevention and control of diseases is chaired by either the Ministry of Health, a Deputy Prime Minister,^8^ or the Prime Minister. Meanwhile, local steering committees on the prevention and control of diseases are chaired by chairmen or chairwomen of the Peoples Committee of the same level who are also the deputy secretaries of the Party Committees at the same level. It can be observed that there are multiple chains of command over a position—chairmen or chairwomen of the Peoples Committees. Practically speaking, as matters of law and politics, the voice of the chair of the National Steering Committee on Prevention and Control of Diseases [except for the case in which the Prime Minister chairs the National Steering Committee] is not quite influential. The reason for this is that (i) the LPCDD does not make clear the legal value of commands of the chair of the National Steering Committee (consequently, the extent to which the voice of the chair is influential is derived from the power of office that the chair holds); (ii) except for the Prime Minister, other positions, e.g. a Ministry of Health, or a Deputy Prime Minister, are not empowered by law to command chairmen or chairwomen of the Provincial Peoples Committee; (iii) as a matter of politics, according to the principle ‘Tập trung dân chủ’ (Democratic centralism)—the key pillar in Vietnamese politics, ‘Party members and bodies have to obey the Party Resolutions. The minority has to obey the majority, bodies at a lower level have to obey ones at the higher level, individuals have to obey the organization…’ (see, for example, Article 9 of the VCP’s Charter), chairmen or chairwomen of the Peoples Committees—the deputy secretaries of Provincial Party Committees must strictly comply with decisions reached by Provincial Party Committees and Provincial Standing Committee of Party Committees after the collective deliberation and voting procedure internal to such committees (as opposed to the Chair of the national steering committee on prevention and control of diseases); (iv) Provincial Party Committees and Provincial Standing Committee of Party Committees are chaired by Party secretaries who can be members of the Central Party Committee or even the Politburo; these persons are no less politically influential—if not to say more influential—than a Deputy Prime Minister, a member of the Central Party Committee. Consequently, the National Steering Committee on the Prevention and Control of Diseases lacks the capacity to supervise and ensure the coherence and consistency of measures.

#### Saving-Face and Oral Orders

‘Saving face’ has long been a widespread practice in Sinosphere countries (Barbalet 2014). ‘Face’ is a sociological-cultural concept that has nuanced implications, including “socio dynamic valuation”, “respect”, and/or “prestige”. To not make a higher-ranking officer lose face, a lower-ranking officer is required to fully obey the orders of the higher-ranking officer even when these orders are just made orally, via phone calls, for example. As shown earlier, under the principle of ‘Democratic Centralism’, collective deliberation and voting internal to the Provincial Party Committees and Provincial Standing Committee of Party Committees are required for adopting a policy or measure, oral orders do not follow this procedure, and as such, can bypass being supervised. Besides, without being recorded in official documents, oral orders might mask and shield the officer making decisions from responsibility.

Unfortunately, the principle of ‘Democratic Centralism’ in requiring a lower-ranking officer to submit to the higher-ranking authority (the lower-ranking officer can only and must report concerns to the supervising body of that higher authority while carrying out the order) can be too demanding to prevent misuse of powers fuelled by saving-face practice.

#### Lacking a Judicial Review or Equivalent Mechanism

Because under the socialist legality, the law is only a product of, and as such, submissive to, the State’s mind (CPV 1960; Nghia and Ha 2018; Tâm 2009), judicial review—the power of the courts to examine the actions of the state, specifically the legislative, executive, and administrative bodies, to determine whether such actions are consistent with the constitution—does not exist.

#### Too Many Documents to Supervise

Empirical research conducted by Le et al. (2021) shows that in the first 6 months of the pandemic (from 13 January 2020 to 24 July 2021), 959 policy documents were issued and new policy documents were issued every single day. A search on Law Library (Thư viện pháp luật)—one of the biggest databases of Vietnamese policy documents—shows that there have been around 2051 documents as of 11 October 2021, the date on which Vietnam declared to return to normalcy in light of Resolution 128/NQ-CP (Resolution 128). Too many documents being promulgated also increases the difficulty in supervision, especially when the central government does not have an idea as to how to simultaneously leave room for the autonomy of local authorities and control such autonomy.

## Recommendations

From all three cases above, it is clear how human rights can be vulnerable during the pandemic. As many scholars argue, it is doubtful that the protection of other human rights should be [blanketly] derogated under the justification of the pandemic (Dzehtsiarou 2020; Frowde et al. 2020; McQuigg 2022) and if the blanket derogation of human rights does add any additional values to the protection of health. From the three cases above, it is also not hard to imagine how abusive and unreasonable measures might harm dignity, physical and mental integrity, and health. The need for human rights and upholding human rights during the pandemic or emergencies, as such, should be recognized. from the above cases and the arguments presented, two solutions are suggested.

### Improving the Supervision of the Decentralization Process

The Vietnamese Government has recognized the lack of supervision over the decentralization and the delegation of power and the fragmentation, divergence, and inconsistency of measures and called for strengthening the supervision (Văn 2021). Accordingly, it passed Resolution 128/NQ-CP (Resolution 128) issuing regulations on “safety, flexibility, and effective control of the COVID-19 epidemic”, whose aims are to establish new normalcy and unifying measures across the nation. Resolution 128 suggests a two-layer mechanism for supervision: *first*, by emphasising the leadership of the Party, it calls for the engagement of the local Party Committees and Provincial Standing Committee of Party Committees in supervising measures made by administrative bodies at the same level; *second*, it requires that if a local authority wants to invent a measure that is more intrusive than Government’s measures, it must get permission from an upper authority. Notwithstanding, it seems that Resolution 128 is more about a political message that reminds local authorities to stop drastic and divergent measures because such measures are no longer needed than a technical legal solution. The reasons supporting this claim are that (i) when Resolution 128 was promulgated, a large portion of the Vietnamese population had been vaccinated, infections and risks were significantly reduced; (ii) Resolution 128 cannot suspend divergent measures outright, and such measures were dropped gradually by local authorities later; (iii) the practical enforcement of Resolution 128 has never been observed.

Besides, there are reasons to believe that the supervision mechanism launched by Resolution 128 is just a bandage solution and if a public health emergency emerges, it shall not work.

*First*, the supervision mechanism launched by Resolution 128 has not been institutionalized in law. Resolution 128 is only a specific solution at the end of the COVID-19 pandemic and authorities are not bound to follow it in other emergencies.

*Second*, even if the Government replicates the Resolution in the future in response to other emergencies, the supervision mechanism shall fail because the criteria for which an upper authority might permit a lower authority to restrict human rights is not transparent. Moreover, it is quite nonsensical that an upper authority that delegates its power to a lower authority and that might have the same or similar motivations to restrict human rights disproportionately as those of a lower authority is also charged with permitting the lower authority to restrict human rights.

To improve the supervision of the decentralization process, first, the supervision mechanism must be institutionalized; second, the supervision mechanism must be transparent which means criteria for permitting measures that restrict human rights must be articulated explicitly and clearly and reasons justifying any permission must be accessible to the public.

### Improving the Understanding of Human Rights

Awareness and understanding of human rights are pivotally important to ensuring human rights protection. In the Preamble of the Universal Declaration of Human Rights (UDHR), it is read that ‘… [the] Universal Declaration of Human Rights as a common standard of achievement for all peoples and all nations, to the end that every individual and every organ of society, keeping this Declaration constantly in mind, *shall strive by teaching and education to promote respect for these rights and freedoms…*’. Several reasons support improving the understanding of human rights.

*First*, supervision is always insufficient or slower than measures. It is simply not realistic to oversee and fix a huge number of problematic measures and breaches, especially in the implementation of measures.

*Second*, however well-structured a mechanism is, who runs that mechanism does matter too. This is evidence in the fact that more than once judicial review overlooks human rights, to name a few: Dred Scott v. Sandford, Buck v. Bell, Korematsu v. the United States. It is unrealistic to expect an authority who despises or has an inadequate understanding of human rights to safeguard human rights.

*Third*, it is always better if human rights violations have never happened because no compensation can be duly and fully remedy damage and loss caused by breaches.

*Fourth*, as shown in the three above cases, some breaches are a sort of banality that can be prevented by means of raising awareness.

## Conclusion

By studying three cases concerning public health measures, this paper gives a sense of how controversial public health measures made and implemented decentrally might be concerning human rights. It is argued that several social, legal, and pollical factors, including poor understanding of human rights, the undefined breadth of discretion, and lack of supervision, are the underlying factors for such problematic decentralized measures. Accordingly, this paper proposes two solutions (i) improving the supervision of the decentralization process, and (ii) improving the understanding of human rights.

## Data Availability

Not applicable.

## Footnotes

1 According to the Siracusa principles as envisaged under Article 12 of ICCPR and developed at the Siracusa Conference, state actions that limit human rights and freedoms must be:

- In accordance with the law;
- Based on a legitimate objective;
- Strictly necessary in a democratic society;
- The least restrictive and intrusive means available; and.
- Not arbitrary, unreasonable or discriminatory.

2 For example, in the case X, Y, and Z v. The UK, the ECtHR holds that ‘… since the questions raised by the case touch on areas where there is no clear common ground among the Member States, the Court considers that the margin of appreciation to be afforded to the respondent State must be a wide one' (Para. 44).

3 ‘The State shall … recognize, respect, protect and guarantee human rights and citizens’ rights…’.

4 ‘1. In the Socialist Republic of Vietnam, human rights and citizens’ rights in the political, civil, economic, cultural and social fields shall be recognized, respected, protected and guaranteed in accordance with the Constitution and law. 2. Human rights and citizens’ rights may not be limited unless prescribed by a law solely in case of necessity for reasons of national defense, national security, social order and safety, social morality and community well-being’.

5 That means the emergency powers have been used without the formal enactment of the state of emergency. The decision of the Government is driven by two factors: *As a matter of policy,* the Government worried that declaring ‘the State of Emergency’ might cause public fear thereby embroiling public order and escalating the situation. *As a matter of law,* in line with the Soviet legal tradition, the [Vietnamese] state has seen the law as a mere tool of the ruling class, embodying the mind of such class (Duẩn 1980; Tâm 2009). As a corollary, the law can be flexible to cope with policy needs promptly without the need of resorting to extreme measures whose ramifications would be hard to predict. In fact, globally, there are many other countries which take the same approach as Vietnam (Dimitrovs 2020; Dzehtsiarou 2020; Kovács 2020).

6 Article 46. Establishment of anti-epidemic steering committees; Article 47. Epidemic declaration and reporting; Article 48. Organization of first aid and medical examination and treatment; Article 49. Organization of medical isolation; Article 50. Sanitation, disinfection and sterilization in epidemic zones; Article 51. Personal protection measures; Article 52. Other anti-epidemic measures to be taken during an epidemic; Article 53. Control of entry into and exit from class-A epidemic zones; Article 54. Measures to be applied in a state of emergency in case of epidemic; Article 55. Mobilization and requisition of resources for anti-epidemic activities; Article 56. International cooperation in anti-epidemic activities.

7 The Government, however, does not stipulate what ‘essential’ means.

8 Usually, it is the Deputy Prime Minister in charge of supervising the Ministry of Health.
